# A retrospective study of clinical usage of quetiapine XR and quetiapine IR in outpatients with schizophrenia in Denmark

**DOI:** 10.1002/hup.2254

**Published:** 2012-09-20

**Authors:** Charlotte Emborg, Teresa Hallerbäck, Leif Jörgensen, Andreas Carlborg

**Affiliations:** 1 OPUS KlinikkenRisskov, Denmark; 2 AstraZeneca NordicSödertälje, Sweden; 3 Department of Clinical Neuroscience, Karolinska InstitutetStockholm, Sweden

**Keywords:** schizophrenia, quetiapine extended release, quetiapine immediate release, naturalistic study, outpatients, treatment choice

## Abstract

**Objectives:**

The atypical antipsychotic quetiapine is a first-line treatment for schizophrenia. This non-interventional study (NCT01212575) evaluated the clinical use of its two formulations, extended release (XR) and immediate release (IR), in outpatients with schizophrenia spectrum disorder.

**Methods:**

Patients who had received at least one dose of quetiapine XR and/or IR were included. A dosage ≥400 mg/day was defined as antipsychotic. Medical records data were collected retrospectively.

**Results:**

Of 186 enrolled patients, 99 (53%) and 87 (47%) received quetiapine XR and IR, respectively. Use in antipsychotic dosage was seen for 89% XR versus 63% IR patients (mean daily dose ≥400 mg/day; *p* < 0.0001). 75% XR and 53% IR patients used dosages ≥600 mg/day (*p* = 0.0019). Quetiapine XR was used at higher mean daily dosages than IR (748 vs 566 mg/day; *p* = 0.006). Forty-three patients (23%) used both formulations concomitantly; 55 patients (30%) used either XR or IR. Quetiapine IR was used as-needed in 44 patients (23%); one patient used XR as-needed.

**Conclusions:**

Quetiapine XR was used more often in higher (antipsychotic) dosages; quetiapine IR more frequently on an as-needed administration basis. Concomitant use was seen. These findings probably reflect the different profiles of XR/IR and advocate the need for both formulations to offer treatment choice. Copyright © 2012 John Wiley & Sons, Ltd.

## INTRODUCTION

Schizophrenia is a severe and debilitating mental disorder with a worldwide incidence of over 1%. Improving outcome for these patients is essential. However, schizophrenia is difficult to treat for reasons that include the complexity of its pathology (Archer, 2010), a high number of treatment-resistant patients (Hellewell, 1999) and non-adherence to medication (Valenstein *et al*., 2006; Mitchell and Selmes, 2007).

Quetiapine fumarate is a commonly prescribed, first-line, oral atypical antipsychotic (AAP) for schizophrenia (Riedel *et al*., 2007; Baldwin and Scott, 2009). It exists in two formulations: immediate release (quetiapine IR) and extended release (quetiapine XR) with different pharmacokinetic and pharmacodynamic profiles (Kapur *et al*., 2000; Figueroa *et al*., 2009). According to label, quetiapine IR should be used twice daily for patients with schizophrenia, with achieved target therapeutic dose after 4–5 days of dose escalation. Contrary, quetiapine XR with its once-daily dosing and rapid dose escalation schedule will achieve therapeutically effective doses from day 2 onwards (Peuskens *et al*., 2007; Baldwin and Scott, 2009; Figueroa *et al*., 2009). During initial dose titration, quetiapine XR is associated with less sedation than quetiapine IR (Datto *et al*., 2009). With the complexity and difficulty of treating schizophrenia and the different characteristics of quetiapine XR and IR, a different use of the two formulations when treating patients with schizophrenia in real life clinical practice can be anticipated.

The present study investigated the real life usage of quetiapine XR and IR in an outpatient setting in Denmark by retrospective review of medical records. The primary objective is to evaluate the clinical use of quetiapine XR and quetiapine IR in antipsychotic dosages (defined as ≥400 mg/day) in patients with schizophrenia spectrum disorders.

## METHODS

### Study design and patients

This non-interventional, retrospective, multicenter study (clinicaltrials.gov, NCT01212575) was conducted in 13 outpatient clinics geographically distributed across Denmark. Patients of either sex aged 18–65 years and diagnosed with schizophrenia spectrum disorder (ICD10 diagnosis codes F20, F23.1, F23.2 and F25), who had received at least one dose of quetiapine XR and/or quetiapine IR during the study period (1 April 2009 to 30 September 2010) could be included. Sites/patients having prescription restrictions regarding quetiapine XR or quetiapine IR were not eligible for inclusion in the study, neither were patients who were participating in a clinical trial during this period nor who were being treated in forensic care.

All patients who fulfilled the eligibility criteria were, prior to enrolment, asked to sign a Subject Informed Consent Form, in accordance with Danish data protection and privacy legislation, to allow access to their medical records. Only patients who provided the written informed consent could be included. Patients were enrolled into either the quetiapine XR or the quetiapine IR group. In subjects who had received both formulations simultaneously, the highest dose determined which group the patient was enrolled in.

Data specified according to protocol were collected retrospectively from patient medical records by manual search performed at each study site, and entered into a web-based data capture system. All data were kept anonymous and identified only by an enrolment code.

The study was performed in accordance with ethical principles consistent with the Declaration of Helsinki, International Conference on Harmonisation Good Clinical Practices and the applicable legislation on non-interventional studies. Investigators performed the study in accordance with the regulations and guidelines governing medical practice and ethics in Denmark, and in accordance with currently acceptable techniques and medical expertise. The study protocol was sent to the Danish Medicines Agency.

### Study outcomes

A predefined dose cut-off of ≥400 mg/day was used to assess the primary objective of clinical use of quetiapine XR and IR as primary antipsychotic treatment, respectively. The approved dose range for quetiapine XR is 400–800 mg/day, and for IR the range is 150–750 mg/day. The cut-off of 400 mg was chosen because PET studies show D2 receptor occupancy by quetiapine throughout the antipsychotic dose range 400–800 mg/day (Kapur *et al*., 2000).

The following secondary outcomes were investigated: patient baseline characteristics; simultaneous usage of quetiapine XR/IR; as needed administration of quetiapine XR/IR; time since diagnosis; non-pharmacological/psychological treatments; hospitalisations and visits to clinics; usage of concomitant medication; patient comorbidities and reasons for and where treatment was initiated and discontinued (hospitals or out-patient clinics).

### Statistical analysis

A statistical analysis plan was written before clean file, and all analyses were performed using s as 9.2 (SAS Institute, Cary, NC). A chi-square test was used to compare categorical variables; a *t*-test was used for comparison of numerical (continuous) variables. The statistical hypothesis was that the groups would have the same average value, and *p*-values for rejecting this hypothesis were calculated. A *p*-value below 0.05 was considered significant.

## RESULTS

### Patient baseline characteristics

One hundred and eighty six patients (95 men, 91 women) were included in the study, of which 99 (53%) received quetiapine XR and 87 (47%) received quetiapine IR as their main treatment. Baseline characteristics were similar for the two patient groups (Table 1).

**Table 1:** Patient baseline characteristics

Variable	Quetiapine XR	Quetiapine IR	*p*-value
ICD10 diagnosis F20, *n* (%)	80 (81)	76 (87)	0.2952
ICD10 Diagnosis F23, *n* (%)	2 (2)	0 (0)		
ICD10 Diagnosis F25, *n* (%)	17 (17)	11 (13)		
Age years, (SD)	37.6 (13.2)	39.1 (11.5)	0.4129
Females, %	56	44	0.4509
BMI, kg/m^2^	29.2 (8.1)	31.1 (7.9)	0.1456
Education, years (SD)	10.4 (2.6)	10.4 (2.3)	0.9369
Hospitalizations during study period for psychiatric reasons, *n* (SD)	2.64 (5.7)	2.5 (2.2)	0.8985
Own home, *n* (%)	88 (89)	71 (82)	
Sheltered housing, *n* (%)	8 (8)	13 (15)	0.3275

SD, standard deviation; BMI, body mass index; XR, extended release; IR, immediate release.

### Differential dosing of quetiapine XR versus quetiapine IR

Quetiapine was used at an antipsychotic dosage (≥400 mg/day) in 88 (89%) of XR-treated patients versus 55 (63%) of IR-treated patients (*p* < 0.0001; Table 2). The stability of the results were confirmed by using a 600 mg/day cut-off limit; 74 patients (75%) for quetiapine XR versus 46 patients (53%) for quetiapine IR; *p* = 0.0019 (Table 2).

**Table 2:** High and low dose quetiapine XR and quetiapine IR usage

Dose cut-off	Quetiapine XR	Quetiapine IR	*p*-value
≥400 mg/day, *n* (%)	88 (89)	55 (63)	<0.0001
≥600 mg/day, *n* (%)	74 (75)	46 (53)	0.0019

XR, extended release; IR, immediate release.

**Figure 1:**
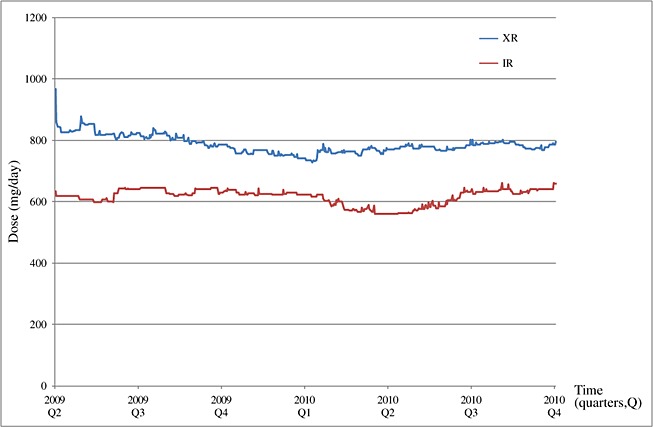
The mean daily dose (mg/day) of quetiapine XR and quetiapine IR versus time (days)

The mean daily dosage of quetiapine XR was significantly higher than that of quetiapine IR during the study period (748 mg/day vs 566 mg/day; *p* = 0.006; Figure 1). In all, 87 patients had once been prescribed a low dosage of quetiapine (≤200 mg/day), excluding the initial titration phase. Among these patients, the mean daily dosage was significantly lower in quetiapine IR than in XR patients (315 mg/day vs 539 mg/day, respectively; *p* = 0.0008).

### Usage patterns with quetiapine XR/IR

Although the majority of patients used either quetiapine XR or IR, a total of 55 (30%) patients used both formulations during the study period, either concomitantly (43 (23%) patients), or sequentially (12 (6.5%) patients; Table 3). Among sequential users, all started with quetiapine IR at a lower daily dosage before switching to a higher daily dosage of quetiapine XR (mean 428 mg/day vs 543 mg/day, respectively).

**Table 3:** Quetiapine XR and quetiapine IR usage pattern during the study period

Type of treatment		Patients, n (%)	Mean XR dose, mg/day (SD)	Mean IR dose, mg/day (SD)
Simultaneous XR and IR	XR in higher doses	30 (16.1)	841 (310)	643 (312)
	IR in higher doses	11 (5.9)	599 (635)	694 (362)
	The same dose	2 (1.1)	400 (282)	400 (282)
Used sequentially, IR before XR		12 (6.5)	543 (230)	428 (239)
Either XR or IR		131 (70.4)	748 (446)	549 (507)
Total		186 (100)	748 (411)	566 (479)

SD, standard deviation; XR, extended release; IR, immediate release.

### As-needed administration of quetiapine XR/IR

The study included a total of 459 prescriptions, of which 45 (9.8%) were specified at an as-needed treatment dosing frequency (Table 4). Of these as-needed prescriptions, 44 were for quetiapine IR and one was for quetiapine XR. Anxiety was the main reason for as-needed prescription (*p* < 0.0001).

**Table 4:** Reasons for and frequency of as-needed quetiapine prescription versus maintenance prescription

Reason	As-needed prescription	Maintenance prescription	Total prescriptions	*p*-value
Sleeping disturbances, *n* (%)	4 (15)	22 (85)	26 (100)	0.3245
Anxiety, *n* (%)	7 (39)	11 (61)	18 (100)	<0.0001
Psychosis, *n* (%)	10 (4.6)	208 (95.4)	218 (100)	0.0003
Schizophrenia, *n* (%)	7 (6.2)	106 (93.8)	113 (100)	0.1327
Miscellaneous, *n* (%)	17 (20)	67 (80)	84 (100)	N/A
Total number of prescriptions, *n* (%)	45 (9.8)	414 (90.2)	459 (100)	N/A

The mean dosage for quetiapine IR when prescribed as as-needed treatment was 152 mg/day. Hospitals were more likely to prescribe as-needed treatment compared with outpatient clinics.

### Time since diagnosis, medication and healthcare contacts

Patients with newly-diagnosed schizophrenia were more likely to receive quetiapine XR than IR (*p* = 0.0009); while there were no significant differences in patient age with respect to XR or IR use (*p* = 0.4129). Patients treated with quetiapine IR as main treatment had been ill for a longer time period than quetiapine XR-treated patients (7.5 (4.3) vs 5.4 (3.9) years (SD), respectively; *p* = 0.0009) and had received less non-pharmacological/psychological treatment during the study period than quetiapine XR-treated patients (46% vs 30% of patients, respectively; *p* = 0.0064).

More scheduled visits were performed by quetiapine XR patients compared with IR patients (19.5 vs 14.4, respectively; *p* = 0.0382); and the number of unattended, unscheduled and psychiatric emergency room visits was numerically lower in the quetiapine XR group (Table 5).

**Table 5:** Patient visits for psychiatric health care

Variable	Quetiapine XR	Quetiapine IR	p-value
Scheduled visits, visits/year	13.0	9.6	0.0382
Unattended visits, visits/year	1.3	1.9	0.1465
Unscheduled visits, visits/year	1.1	1.3	0.7645
Psychiatric emergency room visits, visits/year	0.29	0.45	0.2706
House call visits, visits/year	9.2	10.3	0.5764

XR, extended release; IR, immediate release.

No significant differences were seen with regards to number of hospitalisations, length of hospital stay or ambulatory house call visits.

### Concomitant medication usage

Approximately 80% of all patients were treated with concomitant medications. No difference was seen between the groups with regards to number of concomitant drugs taken (*p* = 0.9344), length of treatment (*p* = 0.9256), or antipsychotic (*p* = 0.7784) or anti-depressive medications (*p* = 0.5486). Further, there was no difference in concomitant treatment for anxiety/sleep disorders (*p* = 0.4405) or mood stabilisers (*p* = 0.5844) between the two groups.

### Patient comorbidities and reasons for treatment initiation and cessation

Comorbid psychiatric medical conditions showed no difference between the groups (*p* = 0.6744). The majority of the patients had no recorded suicidal attempts during the last 2 years, but a total of 11% and 8% in the XR and IR groups, respectively, were reported as having at least one attempt within the same time period (*p* = 0.8353). Only 38 patients had somatic diagnosis reported, and there was no difference between the two groups (*p* = 0.4748). The reasons for treatment initiation and cessation did not differ between the groups.

## DISCUSSION

This study infers a differential use of quetiapine XR and IR in patients with schizophrenia spectrum disorders in a real life outpatient setting. Quetiapine XR was more often used in significantly higher (antipsychotic) dosages than quetiapine IR; and concomitant use of quetiapine XR and IR was seen for almost 25% of the patients, with quetiapine XR most often used at higher doses than IR. Further, a sequential use was also demonstrated, where all patients started on quetiapine IR prior to switching to higher dosages of the XR formulation for the long-term treatment. Taken together, these findings indicate that psychiatrists in an out-patient clinical practice see the two quetiapine formulations as complementary medications rather than substitutes.

Further, newly-diagnosed patients and patients with a shorter disease history were more likely to receive quetiapine XR as primary antipsychotic. They also received more non-pharmacological/psychological treatment during the study period, which follows the recommendations for treating newly-diagnosed schizophrenia in Denmark (Petersen *et al*., 2005). In addition, more quetiapine XR-treated patients were found to attend their scheduled visits to outpatient clinics than quetiapine IR patients, which not only allows the treating physician to monitor the patient health status but also indicate that less chronic patients overall receive more motivational attention for treatments.

The differential use of quetiapine XR/IR seen in this study might be explained by their different pharmacokinetic and pharmacodynamic profiles and reflects their respective titration schemes (Baldwin and Scott, 2009; Figueroa *et al*., 2009). Patients using quetiapine IR were maintained on lower daily doses over a longer period of time, whereas patients with newly-diagnosed or acute schizophrenia were treated by quetiapine XR with its rapid titration scheme (Meulien *et al*., 2010; Riedel *et al*., 2011). As the sedation profile during initial titration of quetiapine IR is different to that of quetiapine XR (Datto *et al*., 2009), it may explain why quetiapine IR was used at lower doses, and as as-needed medication, for its sedative and/or anxiolytic effects (Kasper *et al*., 2004: Philip *et al*., 2008), as well as for additional control of symptoms such as anxiety and psychosis. Interestingly, this treatment was more likely to be initiated in hospitalised patients than in outpatients, and might thus illustrate the clinical need for added medication during exacerbations that require hospital admission.

The subgroup of patients who were treated with concomitant medication, approximately 80% of the study population, was much larger than the elsewhere reported 30–50% (Broekema *et al*., 2007; Wolff-Menzler *et al*., 2010; Barnes and Paton, 2011). Patients with schizophrenia represent a very difficult patient population affected by lack of insight, resistance to treatment and low adherence to medication (Hellewell, 1999; Mitchell and Selmes, 2007). Monotherapy with AAPs is the recommended first-line treatment (Buchanan *et al*., 2010), but as different receptor binding properties result in different efficacy and tolerability profiles, no single AAP is suitable for all patients (Naber and Lambert, 2009; Johnsen *et al*., 2010), and the needed treatment variety leads to individualised patient treatment and eventual polypharmacy (Bingefors *et al*., 2003; Kroken *et al*., 2009; Wolff-Menzler *et al*., 2010). This prescribing behaviour is difficult to study in randomised controlled trials because of the select patient populations and treatment strategies, which do not reflect the full spectra of disease severity and/or comorbidities in a wider population (Simes, 2002; Gorwood, 2006).

A number of non-significant differences between the treatment groups were found in the present study, for example, in relation to use of concomitant drugs. This might be explained by the rather limited number of patients included, as the eventual differences that may prevail between the treatment groups likely would require a larger material to show significance. Further, the non-significant difference seen in relation to healthcare contacts might also be explained by an overall low likelihood of hospital admission as the study population mainly was managed in an outpatient setting.

No significant difference in attempted suicide (11% vs 8%) was seen between the groups based on data from the last 2 years. Compared with what is reported elsewhere on patients with schizophrenia, with approximately 30% documented suicidal attempts from a lifetime perspective and a 5% lifetime suicide risk, this is a rather high figure attempted suicides considering the short study period (Palmer *et al*., 2005; Pompili *et al*., 2007).

This study was designed as a naturalistic study, and the retrospective data collection from medical records prevented any influence on the choice of treatment for patients and produced real life data supporting the hypothesis that ‘no single drug fits all’ (Altamura *et al*., 2008).

The prescribing patterns between the centres were similar, indicating a homogenous prescribing pattern amongst physicians in outpatient care in Denmark and contradicting previous reports of variable prescribing (Bingefors *et al*., 2003; Kroken *et al*., 2009). As expected, both quetiapine XR and IR treatment were initiated more often in district psychiatric clinics (and ambulatory psychiatric clinics for quetiapine XR patients) because only patients with acute exacerbation of their schizophrenia illness are admitted to hospital in Denmark.

This study comes with limitations. First, quetiapine XR was a relatively new drug at the time of study, which may have influenced the results. Also, all antipsychotic medication prescribed only for newly-diagnosed schizophrenia classified as ICD10 F20 (as were the majority of patients, 84%) is free for the first 2 years in Denmark (Birk Andersen, 2011), which may have influenced the prescription behaviour of physicians and their treatment choice and patient adherence to treatment. The price difference between the quetiapine formulations is neglectable. Quetiapine XR is commonly used as first-line in patients with newly-diagnosed schizophrenia or drug-naive patients and especially the young or substance abusers, whereas quetiapine IR is used at lower doses as add-on therapy for comorbid symptoms, including agitation and sleep disorders (Philip *et al*., 2008).

The prerequisite of informed patient consent for enrolment may also have influenced the patient population. More patients may have been enrolled if their informed consent had not been required because many, otherwise eligible, patients were psychotic and especially patients with paranoid symptoms declined participation. This may have affected the ability to show significance for some outcomes and may have created a bias towards patients with milder and less paranoid schizophrenia to be included in the study.

## CONCLUSIONS

In the clinical outpatient setting in Denmark, quetiapine XR was significantly more likely than IR to be used in higher antipsychotic dosages in patients diagnosed with schizophrenia spectrum disorder, whereas quetiapine IR was given at lower doses and more often ‘as needed’ to treat anxiety or psychoses. Almost 25% of patients were treated with quetiapine XR/IR simultaneously, with quetiapine XR given in higher doses than quetiapine IR. Moreover, younger patients with newly-diagnosed schizophrenia were more likely to be treated with quetiapine XR than IR. These results suggest that no one AAP suits all patients and that both quetiapine XR and IR are necessary for physicians to have a wide treatment choice for patients with schizophrenia in clinical practice.
